# Effect of Early Detection and Treatment on Malaria Related Maternal Mortality on the North-Western Border of Thailand 1986–2010

**DOI:** 10.1371/journal.pone.0040244

**Published:** 2012-07-18

**Authors:** Rose McGready, Machteld Boel, Marcus J. Rijken, Elizabeth A. Ashley, Thein Cho, Oh Moo, Moo Koh Paw, Mupawjay Pimanpanarak, Lily Hkirijareon, Verena I. Carrara, Khin Maung Lwin, Aung Pyae Phyo, Claudia Turner, Cindy S. Chu, Michele van Vugt, Richard N. Price, Christine Luxemburger, Feiko O. ter Kuile, Saw Oo Tan, Stephane Proux, Pratap Singhasivanon, Nicholas J. White, François H. Nosten

**Affiliations:** 1 Obstetric Department, Shoklo Malaria Research Unit, Mae Sot, Thailand; 2 Faculty of Tropical Medicine, Mahidol-Oxford Tropical Medicine Research Unit, Bangkok, Thailand; 3 Centre for Tropical Medicine, University of Oxford, Oxford, United Kingdom; 4 Division of Infectious Diseases, University of Amsterdam, Amsterdam, Netherlands; 5 Global Health Division, Menzies School of Health Research, Darwin, Australia; 6 Child and Reproductive Health Group, Liverpool School of Tropical Medicine, Liverpool, United Kingdom; Menzies School of Health Research, Australia

## Abstract

**Introduction:**

Maternal mortality is high in developing countries, but there are few data in high-risk groups such as migrants and refugees in malaria-endemic areas. Trends in maternal mortality were followed over 25 years in antenatal clinics prospectively established in an area with low seasonal transmission on the north-western border of Thailand.

**Methods and Findings:**

All medical records from women who attended the Shoklo Malaria Research Unit antenatal clinics from 12^th^ May 1986 to 31^st^ December 2010 were reviewed, and maternal death records were analyzed for causality. There were 71 pregnancy-related deaths recorded amongst 50,981 women who attended antenatal care at least once. Three were suicide and excluded from the analysis as incidental deaths. The estimated maternal mortality ratio (MMR) overall was 184 (95%CI 150–230) per 100,000 live births. In camps for displaced persons there has been a six-fold decline in the MMR from 499 (95%CI 200–780) in 1986–90 to 79 (40–170) in 2006–10, p<0.05. In migrants from adjacent Myanmar the decline in MMR was less significant: 588 (100–3260) to 252 (150–430) from 1996–2000 to 2006–2010. Mortality from *P.falciparum* malaria in pregnancy dropped sharply with the introduction of systematic screening and treatment and continued to decline with the reduction in the incidence of malaria in the communities. *P.vivax* was not a cause of maternal death in this population. Infection (non-puerperal sepsis and *P.falciparum* malaria) accounted for 39.7 (27/68) % of all deaths.

**Conclusions:**

Frequent antenatal clinic screening allows early detection and treatment of falciparum malaria and substantially reduces maternal mortality from *P.falciparum* malaria. No significant decline has been observed in deaths from sepsis or other causes in refugee and migrant women on the Thai–Myanmar border.

## Introduction

Of all mortality rates none has a greater disparity than the maternal mortality between women living in the least developed countries and those living in industrialized countries. Based on data from 2005, the average lifetime risk of dying from complications related to pregnancy or childbirth was 300 times higher in least developed countries. [1] Global maternal mortality has decreased from 422 (358–505) in 1980 to 320 (272–388) in 1990, and was 251 (221–289) per 100 000 livebirths in 2008. [2] Most of this gain comes from a few countries, such as China and is associated with remarkable economic growth. Much of the reduction in maternal mortality in East Asia is attributed to improvements in obstetric care, and a shift away from home births. [3] In contrast, maternal mortality has increased in other countries, particularly those affected by conflict or high rates of HIV infection, mostly in Sub-Saharan Africa.

Thailand has a population of over 60 million. It has experienced significant improvements in population health and a decline in maternal and child mortality. [4] This has been achieved largely by substantial investment in the district health system since 1975. [5], [6] The relatively low maternal mortality ratio [95%CI] (per 100,000 live births) in Thailand: 50 [31–78], 63 [41–89] and 48 [32–68], in 1990, 2000 and 2008, contrasts with estimates from Thailand’s western neighbor, Myanmar: 420 [23–470], 290 [170–510] and 240 [140–410], in the same years [7].

In rural areas of Southeast Asia malaria transmission of *P.falciparum* and *P.vivax* is low and seasonal. In Prapokklao Regional Hospital in Chantaburi on Thailand’s eastern border with Cambodia, in the early 1980s, malaria was the most common cause of maternal mortality. The development of an “action for survival” program dedicated to the care of pregnant women with malaria resulted in a dramatic reduction in maternal deaths from 341 to 54/100,000 live births from 1981 to 1986. [8] In 2008, the malaria attributable maternal mortalities in Thailand and Myanmar were 0.4 [0.1–0.9] and 34 [17–56] per 100,000, respectively [7].

Accurate data are crucial for monitoring progress towards the millennium development goal 5 (MDG), to: “Reduce by three quarters, between 1990 and 2015, the maternal mortality ratio” [9], and MDG 6c: to: “Have halted by 2015 and begun to reverse the incidence of malaria and other major diseases”. Such data are difficult to obtain in resource poor settings where maternal mortality is highest in high risk groups such refugee and migrant populations. [10] Surveys in active conflict zones in eastern Myanmar in 2002–3, suggested a maternal mortality as high as 1,000 to 1,200 per 100,000 live births [8] but causes were not determined. [11] Malaria was undoubtedly a significant contributor: in 2008, 56% of pregnant women surveyed in eastern Myanmar, reported that they had a malaria test (rapid test) in their last pregnancy, of which 12% were positive [12].

The Shoklo Malaria Research Unit (SMRU) is based on the north-western border of Thailand and provides health care to refugee and migrant populations. In response to an estimated malaria maternal mortality from *P.falciparum* malaria of 1% (5/500) in 1985–86 i.e. a single cause equating to a maternal mortality ratio of 1,000 per 100,000 live births, a system of weekly antenatal clinics offering frequent screening for early detection and treatment of malaria was established. [13] This manuscript reports on maternal deaths within that system which has been available to refugees for 25 years (1986–2010) and to migrant women for 12 years (1998–2010).

## Methods

### Ethics Statement

Permission from each individual woman for their information from pregnancy records to be stored and used for research was not obtained because the records were anonymised. Ethical approval for retrospective analysis of pregnancy records was provided by the Oxford Tropical Research Ethics Committee (OXTREC 28–09).

### Study Area and Population

**Figure 1:**
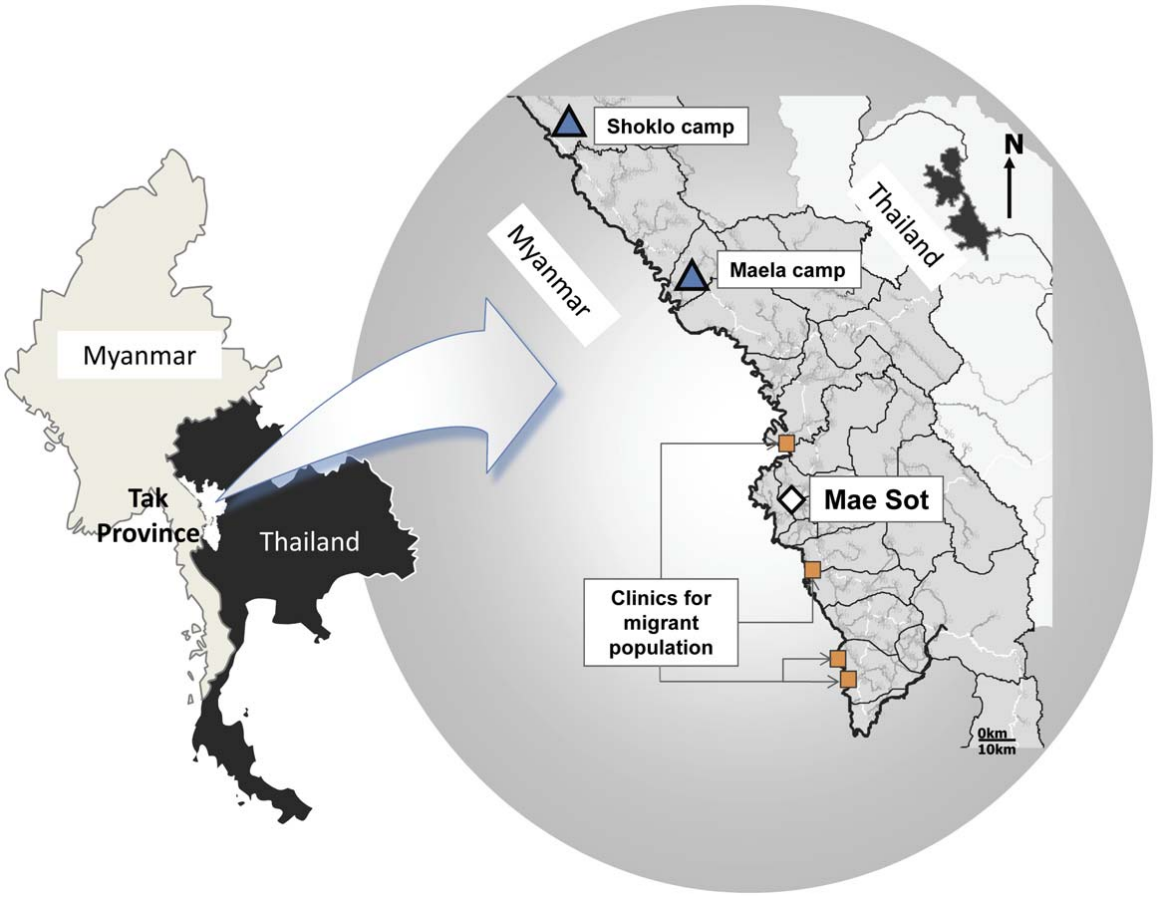
Study area sites of migrant and refugee clinics on the Thai-Myanmar border. Sites for migrants denoted by circles (orange) and refugee by squares (blue).

The SMRU is situated on the north-western border of Thailand (Figure 1). Malaria transmission is low and seasonal in this area and has been described well elsewhere. [14] Briefly, symptomatic malaria occurs in all age groups and pregnant women are at higher risk of severe malaria than non-pregnant women of the same age. [15] Both *P.falciparum* and *P.vivax* malaria have been shown to reduce birth weight in this setting. [13], [16] In a randomized controlled trial of insecticide treated nets in pregnant women in this area, no significant reduction in malaria episodes was observed [17]–[19]. There are no reliable malaria preventative measures [17]–[19] for pregnant women and treatment options are limited because of a high prevalence of multidrug resistant *P. falciparum* and because primaquine for radical cure of vivax malaria is contraindicated in pregnancy. [20] Pregnant women have been encouraged to attend clinics providing antenatal care (ANC) for the early detection and treatment of malaria since 1986 for refugees and since 1998 for migrants (Figure 1).

Refugee camps were established on the western border of Thailand in 1984 as ethnic Myanmar, mostly of Karen origin, fled armed conflict. Shoklo was formerly the largest camp on the border with a population at its peak of approximately 9,000. In 1995–6, Shoklo and other smaller camps were closed and the population regrouped into Maela camp, currently the largest settlement, with an estimated population of 45,000. Non-government organizations providing education and the majority of healthcare and the Thai-Burma Border Consortium providing food rations and building materials have supported the refugee population. Approximately 5% of women attending antenatal care services in the refugee camp come from surrounding villages. The acute complex emergency conditions of the early years of the refugee camp changed to the more stable, chronic refugee situation of today. The migrant communities are mostly involved in seasonal forest and agricultural work along the border, and women frequently move from the area before the pregnancy outcome is known. Provision of health care on the Myanmar side of the border has been non-existent or uncoordinated and access to the Thai system is limited by language, security, and transportation difficulties.

### Antenatal Care and Delivery

Women are encouraged to attend antenatal care as soon as they are aware of their pregnancy. More than 90% of pregnant women in the camps for displaced persons where SMRU operates (current population 45,000) have attended for regular malaria screening. [13] At the first consultation, an obstetric and medical history is recorded, and a clinical examination performed. At each weekly visit a finger prick blood sample is examined for malaria by trained microscopists. Haematocrit measured every two weeks commenced in 1991. Women are also encouraged to attend if they feel unwell at any time between routine consultations to any of the SMRU facilities which are open 24 hours per day. Malaria and other medical problems are treated free of charge. There is no presumptive treatment of malaria. Women receive thiamine (Vitamin B1, 100 mg daily) to prevent infant mortality from beri-beri [21], and ferrous sulphate and folic acid at prophylactic or treatment doses. Testing for HIV for the prevention of mother to child transmission has been available since 2001 in the refugee population and 2008 in migrants. The seroprevalence of syphilis and HIV remain very low in this area: <0.5% for both [22].

In the early years of the refugee camps, close to 100% of deliveries were at home with traditional birth attendants and only problem cases delivered in SMRU or were referred to the Thai Public Hospital. Delivery with the assistance of trained midwives at SMRU was promoted with increasing effort from 1994 onwards and place of delivery (home, SMRU or Thai hospital) was added to routine data collection. Abortion is prohibited in Thailand and was not provided by medical facilities but some traditional birth attendants are reported to provide unsafe termination practices. [23] Pregnancy dating relied on Dubowitz gestational age (GA) assessment and fundal height before 2001 and was then replaced by ultrasound dating. [24]–[26] Local microbiological services were established in 2008.

SMRU is staffed by locally trained medical assistants, nurses, midwives, sonographers, laboratory technicians, home-visitors, support staff and a small number of expatriate doctors. As just under half of the pregnant women are literate a large component of the work involves verbal explanation using a mosaic of languages, mostly Sgaw Karen, Poe Karen, Burmese and English [27].

Anonymized medical records of refugee and migrant women who attended SMRU antenatal clinics between the 12^th^ May 1986 and 31st Dec 2010, were examined. The primary objective of this work was to determine the trends in maternal death across time, and the causes of death in refugee and migrant women. There were no exclusion criteria of this population based study.

### Cause of Maternal Death

In this study the ICD-10 definition of maternal death was used: “the death of a woman while pregnant or within 42 days of termination of pregnancy from any cause related to pregnancy or its management, but not from accidental or incidental causes”. [28] Direct maternal deaths were defined if obstetric related, indirect deaths if aggravated by pregnancy and incidental deaths if unrelated to pregnancy. The maternal mortality ratio (MMR) was defined as the number of maternal deaths (direct and indirect) per 100,000 live births. Incidental deaths were not included in maternal mortality calculations [2].

Autopsy was not available whether death occurred at SMRU or in the Thailand referral hospital. In the case of a death at home, interview for a verbal autopsy with a close family member/s, and where possible the traditional birth attendant present at the time of death, was sought. Assignment of the cause of death was based on collation of the medical and obstetric data from the records, clinical symptoms and signs, laboratory results, and notes from interviews with family members, independently reviewed by 3 doctors (RM, MB, MR). The results were compared and the doctors met to try to resolve the discrepant diagnoses. When resolution was not possible two senior physicians working in the area (FN and NJW) gave the final assignment, if possible.

**Table 1:** Demographic characteristics on enrolment associated with maternal death in refugee and migrant women.

Demographics		Did not die N = 50,910	Died N = 68	Unadjusted Odds Ratios [95%CI]; P value	Adjusted Odds Ratios [95%CI], P value
First ANC visit	Trimester 1	24,108 (50.0)	25 (37.9)	Reference group	Reference group
	Trimester 2 or 3	24,083 (50.0)	41 (62.1)	1.64 [1.00–2.70]; P = 0.051	1.58 [0.91–2.72]; p = 0.101
Weight group	Normal (≥40 kg)	42,630 (93.6)	57 (83.8)	Reference group	Reference group
	Under (<40 kg)	2 911 (6.4)	11 (16.4)	2.83 (1.48–5.39); p = 0.002	**2.76 [1.28–5.92]; p = 0.009**
Age group	<40 years	48,813 (96.1)	62 (91.2)	Reference group	Reference group
	≥40 years	1,987 (3.9)	6 (8.8)	2.38 [1.03–5.50]; p = 0.037	1.41 [0.51–3.75]; p = 0.496
Parity group	Parity 0	14,471 (28.5)	17 (25.4)	Reference group	Reference group
	Parity 1–3	25,619 (50.5)	23 (34.3)	0.76 [0.41–1.43]; 0.401	0.97 [0.48–1.97]; p = 0.932
	Parity > = 4	10,640 (21.0)	28 (40.3)	2.16 [1.18–3.96]; 0.013	**2.22 [1.06–4.68]; p = 0.035**
Anaemic 1^st^ visit	No	24,543 (58.1)	21 (36.8)	Reference group	Reference group
	Yes	17,719 (41.9)	36 (63.2)	2.27 [1.31–3.99]; 0.003	**2.10 [1.20–3.68); p = 0.010**
Limit∧					
Smoker	No	25,927 (69.4)	25 (56.8)	Reference group	Reference group
	Yes	11,452 (30.6)	19 (43.2)	1.72 [0.95–3.13]; 0.100	1.36 [0.71–2.59]; p = 0.356
Migrant	No	24,424 (63.4)	22 (50.0)	Reference group	Reference group
	Yes	14,128 (36.6)	22 (50.0)	1.73 [0.96–3.12]; 0.082	1.54 [0.84–2.81]; p = 0.164

Missing data: In the Did not die columns - First ANC visit = 2719; weight group n = 5369; age group 110; parity group n = 181; and Anaemic 1^st^ visit n = 8,648. In the Died column First ANC visit  = 2; Anaemic 1^st^ visit = 11. Did not die column for smoker = 1176.

∧Model includes 38,935 women and following “Limit” i.e. data of women who delivered from 1998 onwards, the model includes 34,208.

Malaria was diagnosed based on microscopy of blood slides. Asymptomatic carriage is unusual in this area of low seasonal malaria transmission, and invariably associated with low parasitaemias, so a positive blood slide is highly specific for malaria as the cause of febrile illness. Sepsis was defined as severe non-malaria febrile illness with fever, signs of shock including hypotension, tachypnoea or anuria These deaths could be further subdivided into confirmed infection with localizing signs or no localizing signs.

### Pregnancy Outcomes

Miscarriage was defined as a pregnancy ending before 28 weeks gestational age and stillbirth as delivery from 28 weeks or ≥800 g birth weight in which the infant displayed no sign of life (gasping, muscular activity, cardiac activity). The 28-week gestation cut-off, rather than the current WHO 22 week cut-off was chosen, as no infant respiratory support is available in the clinics. Preterm births were deliveries before 37+0 weeks gestational age (GA). Severe malaria was defined as *P.falciparum* parasitaemia with signs of severity such as loss of consciousness or multi organ dysfunction as defined by WHO. [29] Symptomatic malaria was defined as any parasitaemia and a history of fever in the last 48 hrs or a measured temperature ≥37•5°C [30] and asymptomatic malaria as parasitaemia without fever or history of fever. Severe anaemia was defined as haematocrit (HCT) <20% and anemia as HCT <30%.

### Statistical Analysis

Data were analyzed in SPSS for Windows version 19.0 (SPSS, Inc., Chicago, Illinois, USA) and STATA version 11 (StataCorp, College Station, Texas USA). Comparisons were made using the Student’s t-test or Mann Whitney U-test. Categorical variables were compared using the chi-squared or Fisher’s exact test. To assess the demographic factors at the first antenatal visit associated with maternal death trimester, age, weight, parity and anaemia were examined for all women by univariate logistic regression analysis. Information on smoking was only gathered routinely from July, 1997, and migrant consultations commenced in 1998 so a 2^nd^ analysis was done including women who delivered from 1998 onwards. Multivariable logistic regression analysis was used to estimate the adjusted odds ratio after accounting for factors significant (P<0.10) in the univariate analysis.

**Table 2:** Causes of direct and indirect maternal death by parity group.

Cause of death	Parity 0 N = 17	Parity 1–3 N = 23	Parity > = 4 N = 28	Total N = 68 (%)
**DIRECT DEATHS (obstetric related)**	**10**	**9**	**13**	**32**
Haemorrhage	6	6	7	19 (27.9)
Stroke	0	0	5	5 (7.4)
Eclampsia	4	0	0	4 (5.9)
Fatal thrombosis	0	1	1	2 (2.9)
Unknown	0	2	0	2 (2.9)
**INDIRECT DEATHS (aggravated by pregnancy)**	**7**	**14**	**15**	**36**
Sepsis	3	7*	5	15 (22.1)
Severe *P.falciparum* malaria	4	4	4	12 (17.6)
Cancer	0	2	2	4 (5.9)
Tuberculosis	0	0	2	2 (2.9)
Medical complications	0	1	1	2 (2.9)
Unknown	0	0	1	1 (1.5)

* includes one patient with HIV.

## Results

Between the 12^th^ May 1986 and 31^st^ December 2010 there were 71 maternal deaths recorded amongst 50,982 women who attended antenatal care at SMRU at least once. Three (4.2%) were from suicide. Two women, both refugees, aged 36 and 19 years, took antimalarials in overdose including quinine and chloroquine and one aged 26 years, drank weed killer. These cases were excluded from all further analyses.

The majority of pregnancies 73.8% (37,612/50,978) were followed until delivery (live- or stillbirth) 8.9% (4,518/50,978) miscarried, and for 17.4% (8,848/50,978) the pregnancy outcome was unknown, usually because the mother left the catchment area. The majority 72.2% (36,827) of the women attended the clinics in the refugee camps and 27.8% (14,151) those for migrant women. Data on whether delivered women had a live- or still-birth were available for 99.4% (37,403/37,612) of the birth records. Live births accounted for 98.6% (36,895/37,402) of births. The estimated maternal mortality ratio (MMR) was therefore 184 (95%CI 150–230) per 100,000 live births over the 25 year period.

Demographic factors at the first consultation associated with maternal death (Table 1) on multivariate analysis were: being underweight (weight <40 kg), anaemic (haematocrit <30%) and having a parity of 4 or more. When the analysis was confined to women who delivered from 1998 (when the data collection of migrants started) onwards, being underweight (AOR 2.89 (95%CI 1.26–6.62)) and anaemic (AOR 2.12 (95%CI 1.13–3.40)) remained significantly associated with the risk of death.

The median [inter-quartile range] number of antenatal visits, which is equivalent to the number of screens for malaria, was significantly lower in women who died compared to those who did not: 7 [1]–[29] *vs* 12 [1]–[38], p = 0.002l. The proportion of women with 4 or less antenatal visits was significantly and independently associated with maternal death for all women: AOR 2.50 (95%CI 1.41–4.43), p = 0.002 and for women who delivered from 1998 onwards: AOR 2.38 (95%CI 1.22–4.64), p = 0.011.

### Causes of Maternal Deaths

There were 47.1% (32/68) direct and 52.9% (36/68) indirect pregnancy related deaths (Table 2). Haemorrhage, sepsis and *P.falciparum* malaria were the 3 leading causes of death across all parity groups. Overall they accounted for 67.6% (46/68) of all maternal deaths (Table 2). None of the deaths in this series were from suspected induced abortion. Three antenatal records did not have enough information to assign a cause of death with confidence although there was enough information to decide whether the cause was direct or indirect. HIV testing was available for 38.2% (26/68) of the women who died and one was confirmed positive 3.8% (1/26) (Table 2).

### Death from Haemorrhage

Death from haemorrhage (N = 19) occurred in the intrapartum or early post-partum period and included five cases of uterine atony, four placental abruptions, four cases of ruptured uterus, three cases of retained placenta, two completed uterine inversions and one hydatidiform mole (before ultrasound was available). Three women who died of haemorrhage had a concomitant febrile illness (all with no obvious focus of infection) but *Escherichia coli* was isolated from a blood culture in one hospitalised case. This woman was a 26 year old, gravidity 3, parity 2, GA 32.0 weeks, referred with antepartum haemorrhage, placental abruption and disseminated intravascular coagulopathy. Of the 12 women who died at home 41.6% (5/12) died from haemorrhage.

### Death from Sepsis

One of the 15 deaths from sepsis was post-partum, with the remaining occurred during pregnancy 46.7% (7/15), and intrapartum 46.7% (7/15). There were 60.0% (9/15) with no localizing signs. The six cases with localizing sign included: one (probable) puerperal sepsis (3 days fever), one ruptured amoebic liver abscess; one sub-xyphoid abscess with two previous abdominal operations; one suspected *Pneumocystis carinii* pneumonia in a woman with tuberculosis and HIV; one dysentery and one case of cholera (during an epidemic). The length of febrile illness was often protracted with a median of 10 [1]–[35] days fever. Only one woman was attending for the first time and most 64.3% (9/14) had attended their routine antenatal care consultation within the week before presenting with sepsis.

### Death from Malaria

Maternal deaths from malaria mostly occurred during pregnancy without signs of labour: 66.7% (8/12). There were 25.0% (3/12) intra-partum and 8.3% (1/12) post-partum. All were *P.falciparum* and none were *P.vivax* or mixed infections. There were 83.3% (10/12) of women with coma (1 recovered from coma but died on day 5 of pulmonary oedema). Two women died from acidosis and acute severe anaemia with cardio-respiratory failure.

The median duration of fever prior to admission was 4 [2]–[30] days. One woman was attending the clinic for the first time and the remaining 11 women were all absent from their routine antenatal care visit the previous week. The period of absence was for a median of 6 [1]–[14] weeks. Nearly three-quarters, 72.7% (8/12) died within 12 hours of admission.

### Death from Eclampsia and Stroke and Thrombosis

Four primigravidae died from eclampsia. Five multigravid women died from fatal stroke, three with confirmed essential hypertension exacerbated by pregnancy and two (both smokers) presented with a clinical picture consistent with sub-arachnoid haemorrhage, of which one was confirmed on referral to the Thai Hospital.

Two women died from suspected fatal thrombosis: one woman had rheumatic heart disease since childhood and atrial fibrillation of 10 years duration and died suddenly at a gestation of 16+5 wks. Another was 33 y.o, gravidity 3, parity 2, who was reportedly well after an uncomplicated normal vaginal delivery and died suddenly while walking, on day 4 post-partum.

### Death from Other Causes

Four women died from cancer including hepatocellular (Hepatitis B positive), large bowel, and lung (two cases one of which was small cell cancer).

Medical complications included: thyroid storm and one case of chronic respiratory illness with an afebrile exacerbation. There were two deaths from tuberculosis.

### Changes in Maternal Mortality between 1986 and 2010

**Figure 2:**
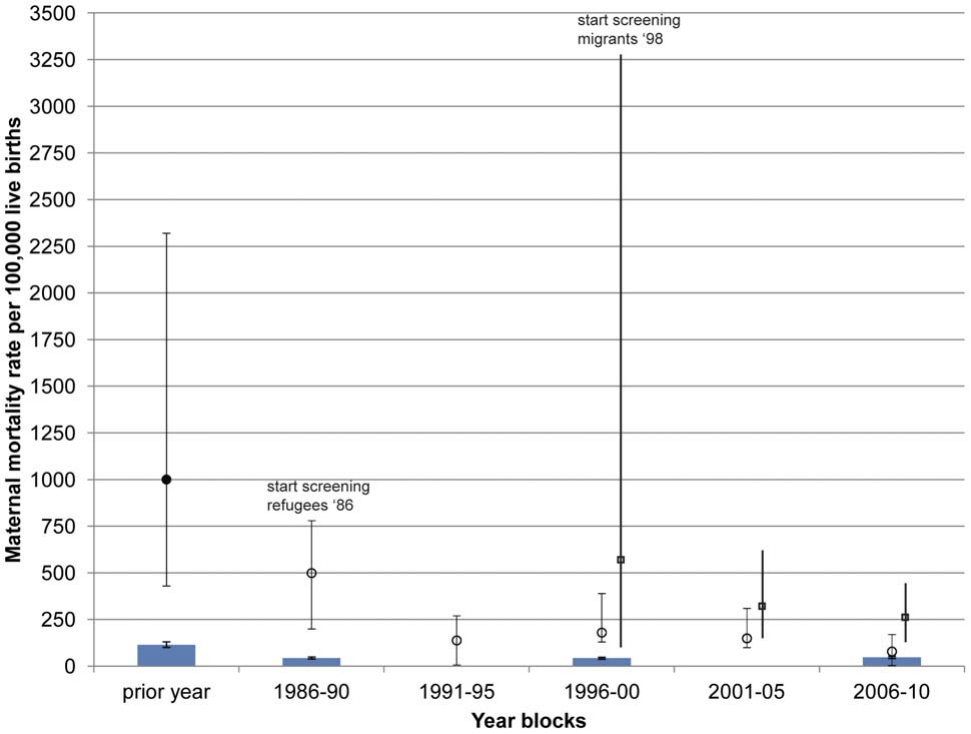
Maternal mortality ratio (95%CI) per 100,000 live births 1986 to 2010. Note the first data point (filled circle) for refugees in the year prior to 1986 is only *P.falciparum* as the sole documented contributor to maternal mortality. Subsequent refugee data (open circles) and all migrant data (open squares) and the 95% confidence intervals (bars) are *all* cause mortalities, summarized for year blocks. Frequent screening and early detection and treatment at antenatal care commenced in 1986 in refugees and in 1998 in migrants. The data for Thailand (*all* cause mortality) is referenced for discrete years 1986, 1990, 2000 and 2008 (blue bars) and the 95% CI are plotted although they are very narrow [32].

**Figure 3:**
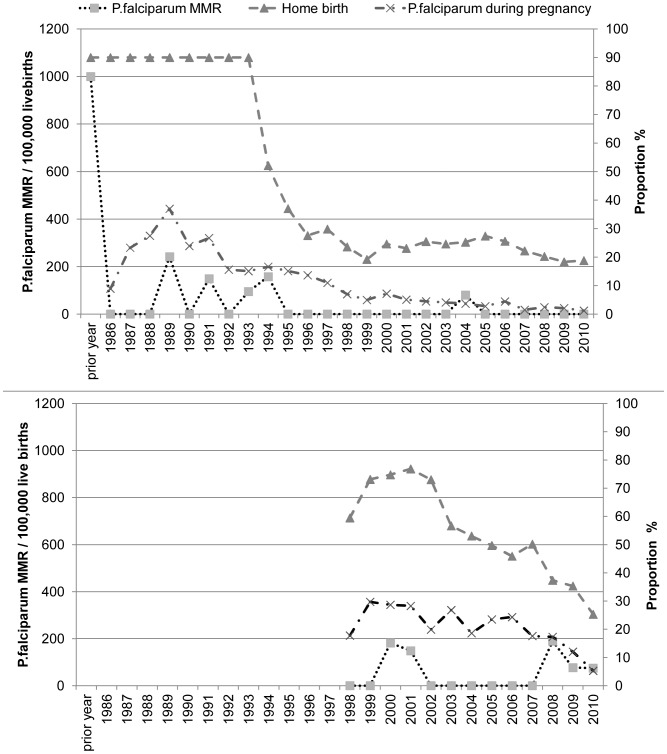
Trends in maternal mortality from *P.falciparum* malaria. **Figure 3a** Refugees: Prior to 1994 the proportion of homebirths was not systematically recorded but was estimated at 90% (right axis). From 1994 the place of birth was systematically recorded and a significant decline can be observed. The fall in maternal mortality due to *P.falciparum* (left axis) occurred with the introduction of weekly screening in May 1986 and before the decline in home birth and the decrease of maternal *P.falciparum* malaria (right axis). From 2005–2010 the proportion of women infected with *P.falciparum* and the proportion of home births have been at their lowest with no maternal deaths in the last 5 years. **Figure 3b** Migrants: Systematic screening in migrant women started in 1998 and the proportion of women with home births (right axis) has reduced markedly as well as the proportion of women with *P.falciparum* infection (right axis). There does not appear to be a relation between home birth and *P.falciparum* related maternal deaths (left axis).

The maternal mortality in refugee and migrant women remains higher than reported for Thailand (Figure 2). There has been a six-fold decline in the overall maternal mortality ratio (MMR) in the refugee camps from 499 (95%CI 200–780) in 1986–90 to 79 (40–170) in 2006–10, p<0.05 (Figure 2). *P.falciparum* malaria related MMR in the refugee camps fell from an estimated 1,000 per 100,000 (430–2,320) [13] in the year prior to the introduction of antenatal screening to zero in 2005 and has remained at that level since (Figure 3a). The sharp fall in maternal mortality due to *P.falciparum* occurred with the introduction of weekly screening in May 1986 and before the decrease in the proportion of women with *P.falciparum* in pregnancy (Figure 3a). From 2005–2010 the proportion of women infected with *P.falciparum* and the proportion of homebirths have been at their lowest levels.

In migrants the 2.3 fold decline in overall MMR from 588 (100–3260) in 1996–2000 to 252 (150–430) for the period 2006–2010 was not significant (Figure 2). There was also a reduction in *P.falciparum* MMR in the migrants from 588 (100–3,260) in 1996–2000 to 78 (30–200) in 2006–10 (Figure 3b). Confidence interval estimates are wide in the early years. *P.falciparum* related deaths in pregnant women in this population before the introduction of the ANC are not available for comparison. The proportion of women delivering at home has reduced markedly amongst migrant women as well as the proportion of women with *P.falciparum* infection (Figure 3b) as in the general population [31], [32]. There is no obvious relation between home births and *P.falciparum* related maternal deaths (Figure 3b).

**Figure 4:**
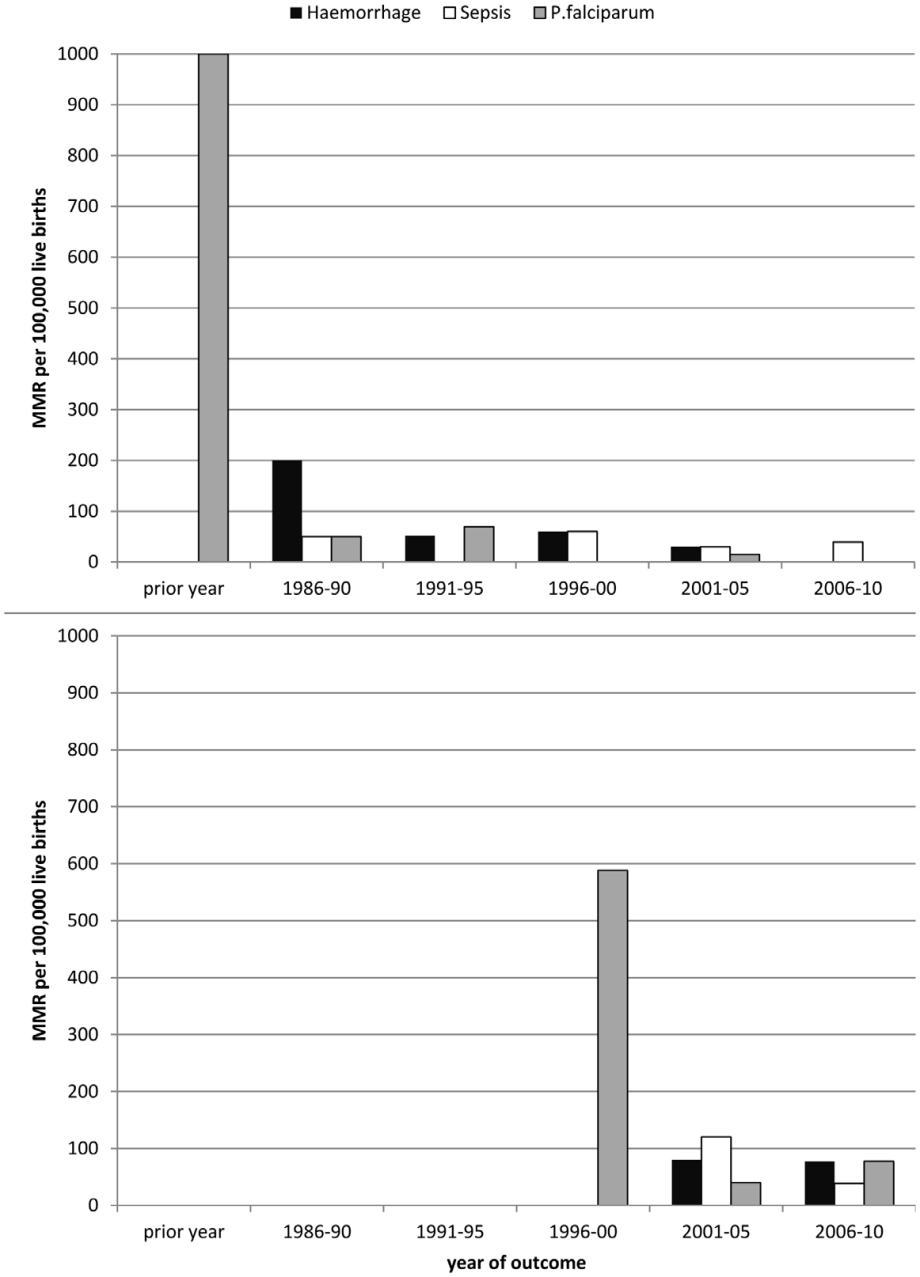
Three major causes of maternal mortality in refugees and migrants on the Thai-Myanmar border. Maternal mortality rates for haemorrhage (black square), sepsis (open square) and *P.falciparum* (grey square) are presented in year blocks for **Figure 4a** refugees and **Figure 4b** migrants. The 1996–2000 year block in migrants represents data collection commencing in 1998.

The maternal mortality of the top 3 causes of death in refugee (Figure 4a) and migrant (Figure 4b) women were plotted by year blocks with no significant differences observed.

### Outcome for the Newborn

There were 32 women who went into labour and delivered before dying, of whom 31.3% (10/32) had a stillbirth, a much higher proportion than amongst the women who did not die, 1.3% (498/37,370); a relative risk (95%CI) of 32.2 (15.2–68.0), P<0.001.

The mean gestational age and birth weight of the live born, singletons in women who died compared to those who did not die was: 37.6 [31.9–45.0] (n = 22) vs 39.0 [28.0–45.0] weeks, P = 0.006; and 2657 [1000–4250] vs 2934 [500–5600] grams, p = 0.402.

## Discussion

In a recent review on trends in maternal death in southeast Asia, using cross-national data sources from 1998–2008, the major complications of childbirth were: haemorrhage 32%, other indirect causes 22% (which were proposed to indicate the still-substantial burden of infectious disease within the region and the effects of malaria and HIV), hypertension 17%, other direct causes 10%, abortion 9%, puerperal sepsis 8% and embolism 2%. [33]. In the rural based population described here haemorrhage (27.9%) was also the most common cause of maternal death, but indirect causes were responsible for over half of the deaths (52.9%) and were mainly caused by sepsis and malaria. The decrease in *P.falciparum* related MMR was unrelated to the decrease in home births.

There has been a significant downward trend in pregnancy related deaths on the Thai-Myanmar border with the most significant decrease being in deaths from severe *P.falciparum* malaria. This is not unexpected given the decline previously reported in malaria and malaria related deaths in the general population on the border, but most of the reduction in MMR occurred before the reduction of malaria incidence in the general population. [31] The decline in MMR comes some two to three decades after Thailand made such gains but MMR along the border still remains significantly higher than reported within Thailand. [2], [8] Pregnant women who develop acute *P.falciparum* malaria have little time to obtain appropriate treatment, as the disease may be fulminant. This is reflected in the short fever history times (average 4 days) in fatal cases and the short interval from arrival at the clinic and death. Provision of emergency obstetric care is unlikely to impact upon this type of presentation. [34] Only early detection and treatment of malaria in the general population, and more specifically frequent screening with early detection and treatment in the pregnant population, has had a very significant impact in this area [32]. There is no safe and effective drug that can be offered as chemoprophylaxis against *P.falciparum* and *P.vivax* because of multidrug resistance. While weekly screening and early treatment has had a significant impact in the refugee population, there are still significant rates of malaria related deaths in the more mobile migrant populations. Absenteeism from the weekly screens appears to be the main significant contributor to malaria related maternal death. This differs from previously published literature which suggests that a reduced number of antenatal visits does not affect the overall outcome of pregnancy. [1], [35] Four or less antenatal visits in this setting was significantly and independently associated with maternal death. Assuming 1% is the ‘normal rate’ of maternal death from malaria without weekly screening in this area and 50,981 pregnant women have been followed up in ANC at least once in the 25 years then approximately 509 pregnancy related malaria deaths have been averted. Unfortunately, malaria related maternal mortality, a highly preventable illness, remains a significant problem in the South-east Asia region. [36] Insecticide treated bednets are unlikely to be a useful preventive strategy in this population [17]–[19].

Sepsis was one of the leading causes of maternal death. Unfortunately microbiological diagnosis was unavailable for the majority of the study period. Unlike women who died from *P.falciparum* malaria these women were mostly present at their routine antenatal clinic before presenting with the terminal illness. The fevers were generally protracted. One of the deaths in this cohort was reported in a study which offered full blood count, malaria smear, haemoculture, urine culture, rickettsia, leptospirosis and dengue investigations, but no causative agent was identified [18], [37], [38]. More sophisticated testing will be required to elucidate the cause of death in such cases. There has been no trend over time towards a reduction in this cause of pregnancy related death.

Unlike recent reports from Sub-Saharan Africa, HIV is not a major cause of maternal death in this area. [39] Part of the reason for this relates to the very low rate of HIV within the population and the use of HAART for all HIV positive pregnant women since 2001 in the refugee population and 2008 in the migrant women. The culture of the Karen ethnic group from eastern Myanmar [22] is likely to have provided some protection from the HIV epidemic within the country but this may not apply to other rural areas in Myanmar [40].

In contrast to most previously published data no deaths from septic abortion were seen and the rate of puerperal sepsis was also very low. [41] Of note is that induced abortion was not a significant contributor to maternal death in this area as reported for a clinic servicing migrant women in an urban setting in Mae Sot, a distance of just 25 to 60 kilometers from the SMRU clinics [23].

The factors associated with death in this study (body weight less than 40 kg, parity of four or more and anaemia at the first consultation) are all potentially modifiable by appropriate interventions such as family planning, nutritional support programs, and information and education. A limitation of this observational study is definitive data on place of birth for all records as well as smoking data prior to 1998.

In total 17.4% of women were lost from follow-up, after enrolment to antenatal care and before delivery outcome, usually returning to Myanmar. In refugee and migrant women clinics that predominantly provide services to women from Myanmar the maternal mortality ratio is still unacceptably high and most likely underestimated: currently 79 (40–170) and 252 (150–430) per 100,000 live births in 2006–10, respectively. The MMR in the refugee camp is approximately 1.7 fold higher than reported for Thailand 47 (42–53)in 2008. [7] Unlike deaths from haemorrhage that are easily linked to labour and delivery, malaria and sepsis may well remain a hidden cause of maternal death as they mostly occur in women without labour, which decreases the likelihood that the death is recorded as pregnancy related.

Measurement of maternal mortality is seldom undertaken in refugee and migrant women or in the rural tropics and the data presented here is limited by the unavailability of laboratory microbiology and of autopsy. The malaria related deaths were confirmed by microscopy. Sepsis and *P.falciparum* malaria are significant contributors to pregnancy related death on the Thai-Myanmar border. *P.vivax* malaria is not. The causes of maternal mortality are not universal and decisions on how to reduce mortality require robust local evidence. Early detection and treatment of malaria with frequent screening in pregnancy has had a profound impact on malaria related maternal deaths on the north-western border of Thailand.
